# Crystallographic characterization and polymorphism in a calcium-based porphyrinic MOF with biomedical potential

**DOI:** 10.1063/4.0001147

**Published:** 2025-10-27

**Authors:** Christelle N. Dzesse T., Mario Wriedt

**Affiliations:** University of Texas at Dallas, Richardson, Tx, USA

## Abstract

Metal–organic frameworks (MOFs) based on biocompatible metals such as calcium are gaining increasing attention in the biomedical field due to their low toxicity, structural diversity, and potential for drug delivery and photodynamic therapy (PDT) [1]. Despite this, calcium-based MOFs incorporating porphyrinic linkers remain rare in the Cambridge Structural Database (CSD). A recent CSD survey conducted in May 2025 revealed fewer than 10 such structures. Among organic linkers, Tetrakis(4-carboxyphenyl) porphyrin (TCPP) stands out for its strong light absorption, high reactive oxygen species (ROS) generation, intrinsic fluorescence, and ability to coordinate with various metals, features that make it highly effective for synergistic PDT and chemotherapy, especially in the treatment of solid tumors [2, 3, 4]. TCPP-based MOFs, particularly those formed with biocompatible metals like calcium or lanthanum, provide enhanced water solubility, minimize aggregation-induced quenching, and improve biocompatibility, essential characteristics for biomedical applications [1].

In this work, we report the synthesis and characterization of a calcium-based MOF assembled with TCPP under solvothermal conditions. The initially isolated phase crystallized in a monoclinic system, [Ca_2_(OH)_2_(TCPP)] (1), with unit cell parameters a = 35.15 Å, b = 6.65 Å, c = 15.97 Å, β = 96.396°, and a volume of 3715.0 Å^3^. Subsequent syntheses yielded a distinct structure with parameters a = 6.90 Å, b = 23.69 Å, c = 17.02 Å, β = 97.37°, and a volume of 2758 Å^3^, suggesting the formation of a new polymorph. This observed polymorphism, despite identical synthetic conditions, demonstrates the structural sensitivity of calcium–porphyrin frameworks to subtle changes in crystallization dynamics. Notably, the second polymorph exhibited improved water and pH stability, confirmed by PXRD over 12 days in water and 4 days at pH 7.4, making it more appropriate for biological environments. These findings reinforce the potential of calcium– TCPP MOFs as stable, multifunctional platforms for biomedical applications, particularly in cancer therapy where drug loading, biocompatibility, and controlled release are critical.

## References

[1] Jiang, X. et al, J. Mat. Chem. B, 2023, 11, 10706–10716 10.1039/D3TB01592K 37917175

[2] Chen, Y. et al, Biomater. Sci., 2024, 12, 1864–1870 10.1039/D3BM01994B 38411494

[3] Xie, Y. et al, 2023, Adv. Nanobiomed. Res*.*, 2023, 3, 2200136 10.1002/anbr.202200136

[4] Zhao, Y. et al, Chem. Mater., 2018, 30, 7511–7520 10.1021/acs.chemmater.8b02467

